# Centromere Architecture Breakdown Induced by the Viral E3 Ubiquitin Ligase ICP0 Protein of Herpes Simplex Virus Type 1

**DOI:** 10.1371/journal.pone.0044227

**Published:** 2012-09-20

**Authors:** Sylvain Gross, Frédéric Catez, Hiroshi Masumoto, Patrick Lomonte

**Affiliations:** 1 Virus and Centromere Team, Centre de Génétique et de Physiologie Moléculaire et Cellulaire CNRS, UMR5534, Villeurbanne, France; 2 Université de Lyon 1, Lyon, France; 3 Laboratoire d'excellence, Labex DEVweCAN, Lyon, France; 4 Laboratory of Cell Engineering, Kazusa DNA Research Institute, Chiba, Japan; McMaster University, Canada

## Abstract

The viral E3 ubiquitin ligase ICP0 protein has the unique property to temporarily localize at interphase and mitotic centromeres early after infection of cells by the herpes simplex virus type 1 (HSV-1). As a consequence ICP0 induces the proteasomal degradation of several centromeric proteins (CENPs), namely CENP-A, the centromeric histone H3 variant, CENP-B and CENP-C. Following ICP0-induced centromere modification cells trigger a specific response to centromeres called interphase Centromere Damage Response (iCDR). The biological significance of the iCDR is unknown; so is the degree of centromere structural damage induced by ICP0. Interphase centromeres are complex structures made of proximal and distal protein layers closely associated to CENP-A-containing centromeric chromatin. Using several cell lines constitutively expressing GFP-tagged CENPs, we investigated the extent of the centromere destabilization induced by ICP0. We show that ICP0 provokes the disappearance from centromeres, and the proteasomal degradation of several CENPs from the NAC (CENP-A nucleosome associated) and CAD (CENP-A Distal) complexes. We then investigated the nucleosomal occupancy of the centromeric chromatin in ICP0-expressing cells by micrococcal nuclease (MNase) digestion analysis. ICP0 expression either following infection or in cell lines constitutively expressing ICP0 provokes significant modifications of the centromeric chromatin structure resulting in higher MNase accessibility. Finally, using human artificial chromosomes (HACs), we established that ICP0-induced iCDR could also target exogenous centromeres. These results demonstrate that, in addition to the protein complexes, ICP0 also destabilizes the centromeric chromatin resulting in the complete breakdown of the centromere architecture, which consequently induces iCDR.

## Introduction

Centromeres are specialized chromosomal domains responsible for chromosome segregation during meiosis and mitosis. In primates they assemble around tandemly repetitive DNA sequences called alpha-satellite or alphoid DNA, in a complex protein structure that has yet to be fully elucidated. A simplistic model involves the division of this domain into two areas: (i) the central core region or centromeric chromatin, assembled around higher order arrays of tandemly repetitive/type I alphoid DNA; and (ii) the flanking heterochromatic regions, called pericentromeres, which are formed around stretches of repeated monomeric/type II alphoid DNA containing other types of repeated sequences, such as long interspersed element (LINE), short interspersed element (SINE), and long terminal repeat (LTR) retrotransposons (for reviews [1]–[3]). The protein composition of the central region is different between interphase and mitosis. In this model, ‘constitutive’ proteins could be associated with the centromere throughout the cell cycle, including interphase, whereas ‘facultative proteins’ are recruited only during mitosis to assemble the kinetochore, which is the site of microtubule attachment. One of the constitutive proteins is CENP-A, the centromeric histone H3 variant that marks centromeric chromatin [4]–[7]. A particular feature of the chromatin structure of the human core centromere is that it contains interspersed blocks of nucleosomes, which contain histone H3 or CENP-A [8]. In addition to CENP-A, five other constitutive CENPs (CENP-B, -C, -H, -I, and hMis12) were initially described as major components of the human interphase centromere [9]–[12]. Then, another set of 11 interphase centromeric proteins was described (for review [13]). Those proteins were found associated with the CENP-A-containing nucleosomes, and distributed within two major protein complexes called NAC (CENP-A Nucleosome Associated) and CAD (CENP-A Distal) complexes, also named constitutive centromere-associated network (CCAN) or CENP-A–NAC/CAD kinetochore complex ([14]–[21] and for reviews [13], [22]). As such, the central core region, including proteins of the CCAN, serves as the assembly platform for the KMN (KNL1/Blinkin/Spc105p, MIND/MIS12/Mtw1 and NDC80/Hec1) protein network, which is essential for kinetochore-microtubule binding [23], [24].

Herpes simplex virus type 1 (HSV-1) is a persistent neurotropic virus capable of frequent symptomatic or asymptomatic reactivations from latently infected human hosts (for review [25]). HSV-1 is a nuclear DNA virus that hijacks the nuclear environment to enable its optimal replication during lytic infection and probably reactivation from latency. The ICP0 protein is synthesized rapidly after infection and is required for the onset of lytic infection and for reactivation of HSV-1 from latency in a mouse model [26]–[28]. In the nucleus, ICP0 temporarily localizes to several nuclear domains such as promyelocytic leukemia (PML) nuclear bodies (NBs) (also known as ND10), centromeres, and nucleoli [29]–[31]. ICP0 is a RING finger (RF) protein, and an E3 ubiquitin (Ub) ligase activity was demonstrated to be associated to its RF domain *in vitro* and/or *in cellulo* ([32]–[36] and for review [37]). As such, ICP0 induces the proteasomal degradation of several cellular proteins, including constituents of the PML-NBs and centromeres, the catalytic subunit of DNA protein kinase, the CD83 surface molecule of the mature dendritic cells, and the histone Ub ligases RNF8 and RNF168 [30], [38]–[44]. ICP0 also possesses several SUMO interacting motifs (SIM) that confer specificity for the proteasome-dependent degradation of SUMO-conjugated proteins [45]. The ICP0-induced destabilization of interphase centromeres in HSV-1-infected cultured cells prevents the assembly of the kinetochore and the binding of microtubules during mitosis [30]. As a consequence, cells that express ICP0 before entering mitosis become stalled in early mitosis, and eventually suffer premature cell division without chromosomal segregation, leading to aneuploidy [30], [46]. Although the biological significance of ICP0-induced centromere destabilization is unclear, ICP0 is a unique tool for studying centromere structure and the cellular mechanisms of centromere architectural maintenance. Until recently, it was not known whether the cell was able to detect centromeric structural defects during interphase. Using ICP0 as a tool to destabilize centromeres, we recently revealed a previously unreported and unexpected cell response [47]. This response, termed the interphase centromere damage response (iCDR), is characterized by the accumulation at damaged centromeres of three proteins, coilin and fibrillarin of the nuclear domain called Cajal bodies (CBs), and SMN protein of the CB-associated Gemini of Cajal bodies (gems) nuclear domain.

In the present study, we investigated the destabilization of the interphase centromere by ICP0. We show that the structure comprised of proteins of the NAC and CAD complexes is destabilized in endogenous centromeres and human artificial chromosomes (HACs). Study of the centromeric chromatin nucleosome occupancy by MNase digestions in HSV-1 infected and ICP0-expressing cells shows that ICP0 modifies the centromeric chromatin. Overall, the present study demonstrates that the entire centromere architecture is significantly altered by ICP0.

## Results

### NAC and CAD complexes are destabilized by ICP0

**Figure 1:**
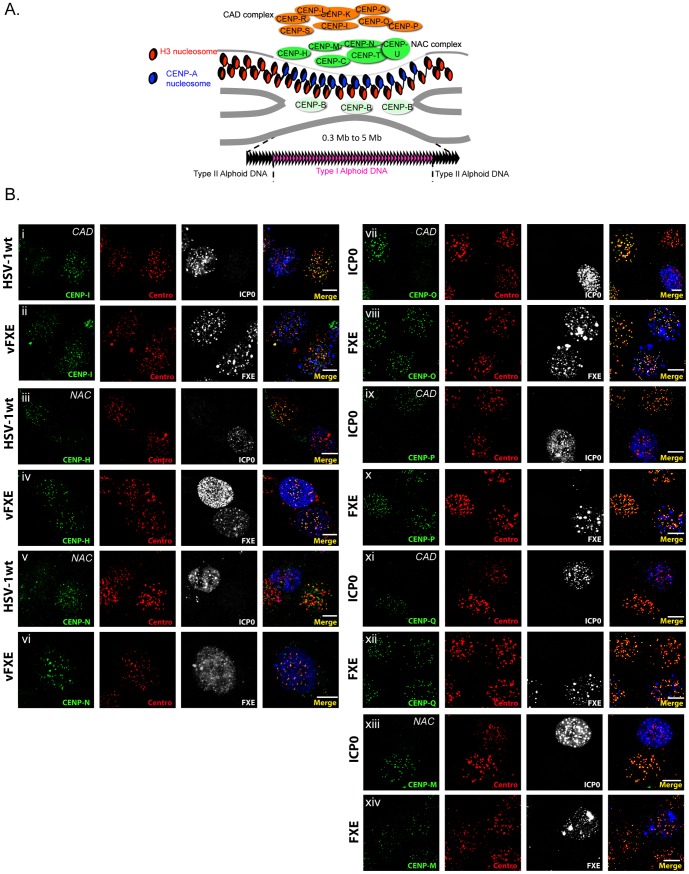
ICP0 destabilizes the entire proteinaceous structure of the centromere. (A) Schematic structure of the interphase centromere. (B) Normal HeLa cells (CENP-I detection) or HeLa cells that constitutively express GFP-tagged CENPs (CENP-H, -N, -M, -O, -P, and -Q) were either infected for 4 h with wild type HSV-1 (HSV-1 wt) or a mutant virus that expresses FXE (vFXE) (i to vi) or transfected with plasmids that express ICP0 or its RING finger mutant FXE (vii to xiv). Detection of CENP proteins, ICP0, and centromeres (centro) was performed by immunofluorescence. The experiment has been repeated several times, and the images faithfully represent what was observed in the overall cell population. Bars, 10 µm.

Interphase centromeres are complex structures that are composed of a particular chromatin that contains CENP-A nucleosomes associated with two major protein complexes initially named NAC and CAD (Figure 1A). To determine whether ICP0 affects the stability of these complexes, we infected or transfected cells and assessed the behaviors of specific NAC- or CAD-associated CENPs in the presence of ICP0, by immunofluorescence and confocal microscopy. HeLa cell lines constitutively expressing a functional GFP-tagged CENP [14] were used to analyze CENP-H, -M, -N, -O, -P or -Q, whereas endogenous CENP-I was detected by IF. Infection by HSV-1 wild type (wt) led to the depletion of all the CENPs analyzed (CENP-I, -H, and –N are shown as examples; Figure 1B, i to vi). In contrast, when cells were infected with the virus that expressed a non-functional ICP0 mutated in its RING finger/E3-Ub ligase activity-associated domain (vFXE), the CENPs were detected as in non-infected cells. To confirm that the observed outcome was due to the activity of ICP0 and not to another viral factor(s), we expressed ICP0 only or the mutant protein FXE by transfection. The expression of ICP0 alone induced disappearance of the CENPs (CENP-M, -O, -P, and -Q are shown as examples; Figure 1B, vii to xiv), whereas the FXE protein did not. These data show that ICP0 is able to induce the removal from the centromeres of CENPs that are associated with either the NAC or the CAD complex.

### ICP0 induces proteasomal degradation of NAC- and CAD-associated CENPs

**Figure 2:**
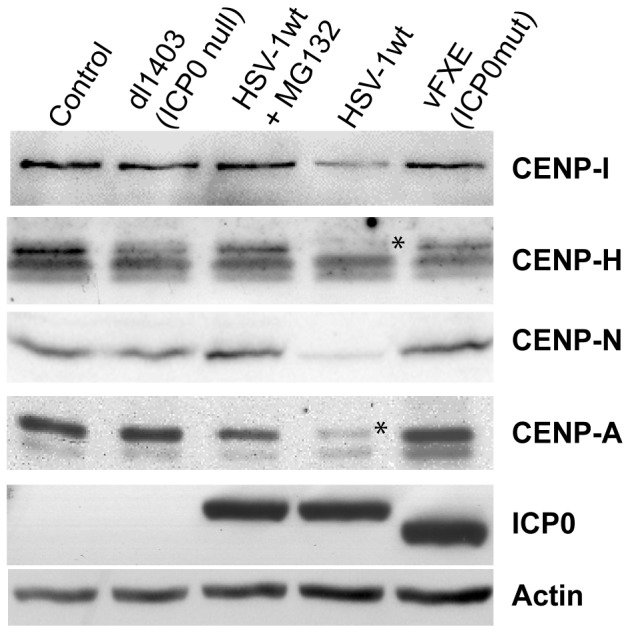
ICP0 induces proteasomal degradation of CAD- and NAC-associated proteins. Western blot detection of CENP proteins from NAC, CAD complexes, and centromeric chromatin in HeLa cells infected for 6 h (m.o.i. of 10) with HSV-1 wt in the absence or presence (+MG132) of proteasome inhibitor, with ICP0-deleted mutant virus (dl1403, ICP0-null) or with mutant virus expressing FXE (vFXE, ICP0-mut). ICP0 and actin are shown as controls for the infection and protein loading, respectively. Stars point out the specific signal corresponding to CENP-H and CENP-A.

To verify whether the disappearance of the signals corresponding to the various CENPs was the result of protein degradation, cells were infected with HSV-1 wt, in the presence or absence of MG132, or with the ICP0 mutant viruses vFXE and dl1403 (ICP0 deleted). Only the CENPs for which we had antibodies suitable for use in Western blots were analyzed. We found that CENP-I (CAD-associated), CENP-H and -N (NAC-associated) were degraded in an ICP0- and proteasome-dependent manner (Figure 2). CENP-A, which is known to undergo ICP0-induced degradation [41], was used as a control. Based on these results and the IF data, we conclude that ICP0 disrupts the entire proteinaceous complexes associated with interphase centromeres.

### Centromeric chromatin structure is disrupted by HSV-1 infection

**Figure 3:**
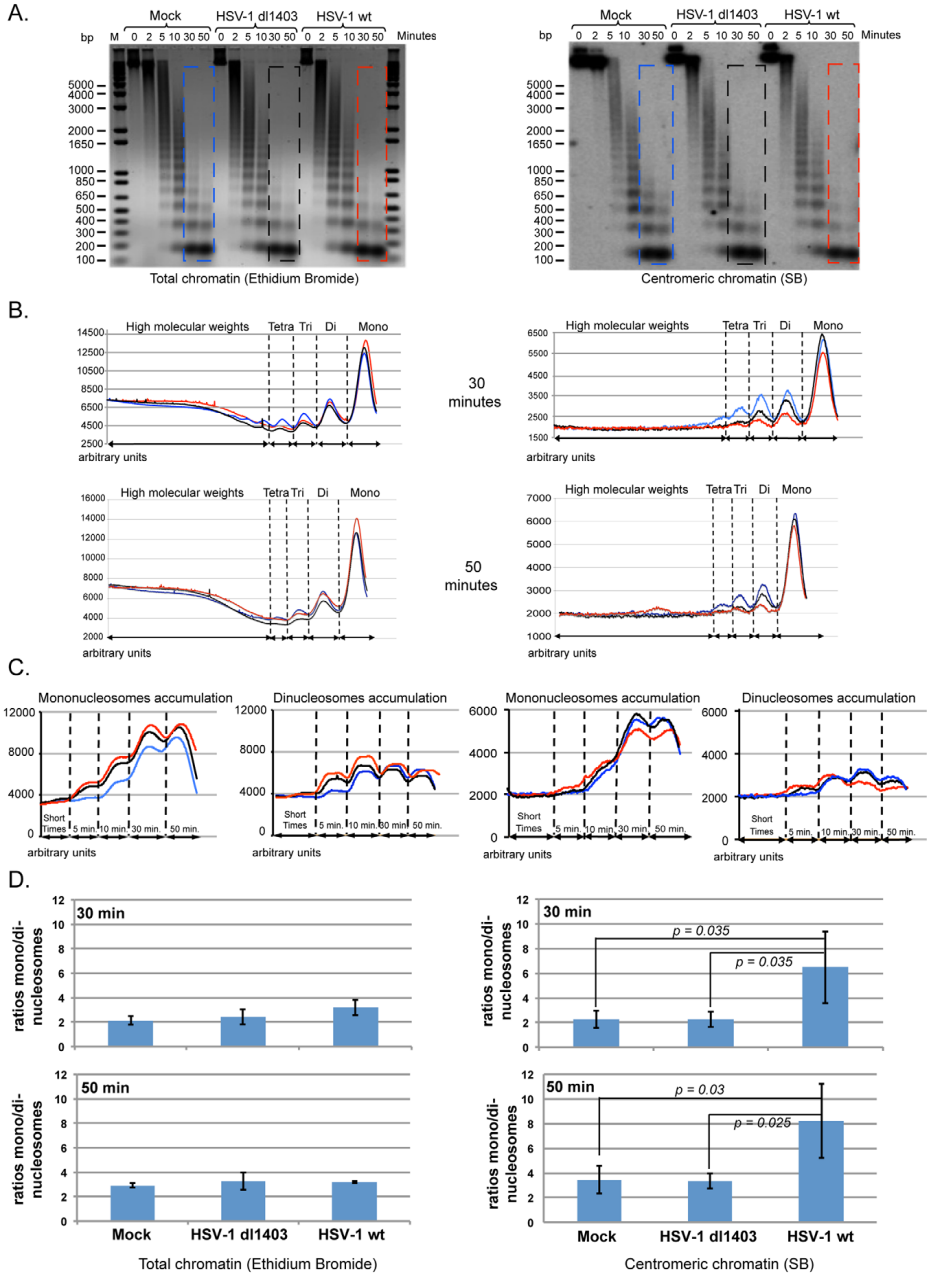
HSV-1 infection destabilizes the structure of centromeric nucleosomes in an ICP0-dependent manner. (A) Micrococcal nuclease (MNase) digestion patterns of the chromatin from HeLa cells subjected to: mock infection; infection with the ICP0-null HSV-1 mutant virus (dl1403); and infection with HSV-1 wt (17syn+). Left panel: MNase digestion pattern of total DNA stained with ethidium bromide. Right panel: Southern blot hybridization of the gel shown in left panel using an alphoid DNA probe. M = 1 Kb Plus DNA ladder (in base pair, Invitrogen). (B) Plot of the digestion profiles after 30 min and 50 min of MNase digestion (curves corresponding to the colored frames in A). Red curve: HSV-1 wt infection; black curve, HSV-1 dl1403 infected cells; green curve: non-infected cells (C) Quantification of mono- and di-nucleosomes over time of MNase digestion. Red curve: HSV-1 wt infection; black curve, HSV-1 dl1403 infected cells; green curve: non-infected cells (D) Rate of chromatin digestion. The ratios of LMW-normalized mono- over di-nucleosomes (see Figure S1) give the rate of digestion of total chromatin (left panels) or centromeric chromatin (right panels). The graphs represent the mean values of the ratios of mono- over di-nucleosomes ± SD for at least three independent experiments. Results were considered as significant for a p value≤0.05 (Student's *t* test).

The destabilization by ICP0 of the protein layers associated with interphase centromeres and in particular the degradation of the CENP-A and CENP-B proteins suggested that the centromeric chromatin undergoes modifications [41], [42]. To test this hypothesis, we performed MNase digestions and Southern blotting (SB) to analyze centromere chromatin nucleosome occupancy in HeLa cells infected for 3 h with the HSV-1 wt or dl1403 viruses. The digestion profile of the total cell chromatin is shown as ethidium bromide-stained agarose gel before transfer (Figure 3A, left) for comparison with the digestion profile of the centromeric chromatin obtained by SB (Figure 3A, right), and are representative of multiple experiments. The results are also presented in graphs of densitometric profiles below the digestion profiles (Figure 3B and 3C). Each curve corresponds to a specific condition (blue, non-infected; red, HSV-1 wt-infected; black, dl1403-infected); the *y*-axis represents the intensity of each nucleosomal species in the analyzed region (in arbitrary units), plotted against the nucleosomal forms (single condition analysis, Figure 3B). Infection with HSV-1 wt led to slightly higher accumulation of mononucleosomes in total chromatin at 30 min and 50 min time points, as compared to the non-infected cells and cells infected with dl1403 (Figure 3B, left panels). Analysis of the total cell chromatin digestion over time (horizontal analysis) also showed a difference in kinetic appearance of mono- and di-nucleosomes between infected and non-infected cells, suggesting a global modification of the cell chromatin by infection, independently of ICP0 (Figure 3C, left panels). To confirm that total chromatin could be affected by infection, we quantified the appearance of high molecular weight (HMW) nucleosomal forms (octo- to penta-nucleosomes) in the mock, dl1403 and HSV-1 wt infected cells (Figure S1). Results showed that the infection induced an earlier appearance of HMW nucleosomal forms compared to control cells, in an ICP0-independent manner. These data suggest that infection *per se* may loosen the higher-order chromatin structure independently of ICP0. This observation is in accordance with previous works (i) from Monier *et al.* [48] who described the annexation of cellular interchromosomal space during HSV-1 infection; and (ii) from the Schang's laboratory, which showed that HSV-1 infection provokes linker and core histones mobilization [49], [50].

Analysis of centromeric chromatin by SB showed that HSV-1 wt-infected cells displayed a deficit in mononucleosomes together with a deficit of the low molecular-weight (LMW) nucleosomal forms, as compared to the non-infected cells and cells infected with dl1403. Markedly, as compared to non-infected or dl1403-infected cells, HSV-1 wt infection led to substantially faster appearance (5 min and 10 min) of centromeric chromatin mono- and di-nucleosomes, and to a quicker disappearance of mononucleosomes (at 30 min in HSV-1 wt vs 50 min in non-infected and dl1403-infected cells) (Figure 3C, right panels). This suggested that ICP0 induced a faster digestion of centromeric chromatin. To further evaluate the kinetic of MNase digestion we determined the ratio between mono- and di-nucleosomes. Indeed, this ratio increases over time and at each time point is indicative of the progression of the digestion. At any given point, the higher the ratio, the faster the digestion. To prevent interference of loading error between lanes, we normalized the quantity of mono- and di-nucleosomes to the quantity of LMW forms (from mono- to tetra-nucleosomes, see methods) (Figure S2, blue and red bars for normalized mono- and di-nucleosomes, respectively). In total cell chromatin the ratios remained similar in the 3 conditions, although we noticed a slight, but not statistically significant, increase in cells infected with both viruses (Figure S2, left panels). However, centromeric chromatin is characterized by an increase in mononucleosomes and a concomitant decrease in dinucleosomes in HSV-1 wt-infected cells after 30 min and 50 min of digestion (Figure S2, right panels). As a result, the ratios of the mono- over di-nucleosome levels at 30 min (6.5±2.9) and 50 min (8.2±3) were significantly higher in the HSV-1 wt-infected than in the mock-infected (2.2±0.7 and 3.5±1.1 for 30 min and 50 min of digestion, respectively) or dl1403-infected (2.2±0.6 and 3.3±0.6 for 30 min and 50 min of digestion, respectively) cells (Figure 3D, right panels). In comparison the mono- over di-nucleosome ratios remained similar in all conditions for total chromatin (Figure 3D, left panels). These results demonstrate that the digestion of the centromeric chromatin is faster in infected cells provided that ICP0 is expressed.

### Centromeric chromatin structure is disrupted by ICP0

**Figure 4:**
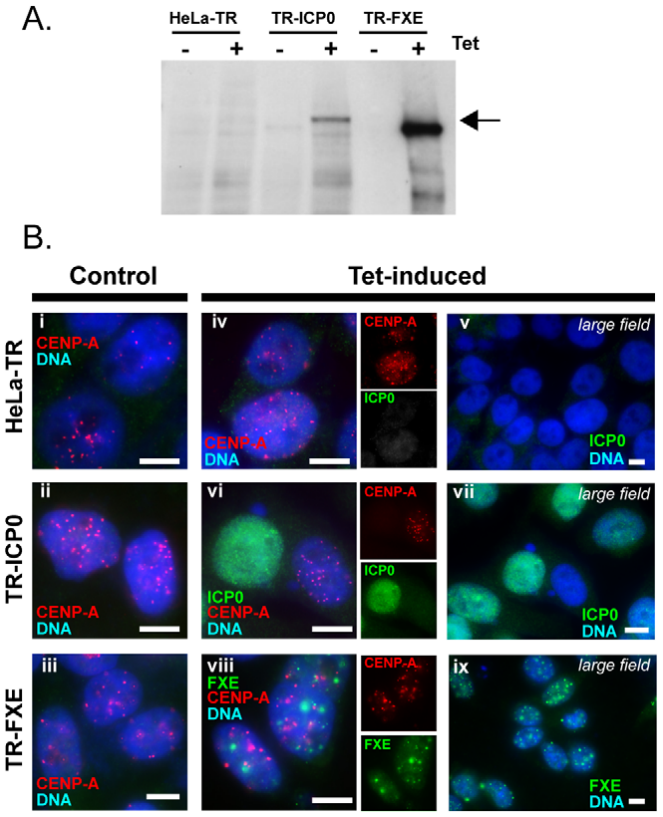
ICP0-expressing cells induce CENP-A loss from centromeres. (A) HeLa-TR, TR-ICP0, and TR-FXE cells were induced (+) or not induced (−) with tetracycline before being subjected to WB to detect ICP0 or FXE expression. The arrow indicates the ICP0 or FXE signal. Note that the level of protein for TR-FXE is 10-fold lower than that for HeLa-TR or TR-ICP0 due to the very high expression of FXE. (B) Control (not-induced) (i to iii) or tetracycline-induced (iv to ix) cells were tested by IF for: (i) the expression of ICP0 or FXE (green); and (ii) the CENP-A (red) signal at centromeres (see insets for individual ICP0, FXE or CENP-A signals). More than 99.5% of the cells are positive for the ICP0 or FXE signal. The CENP-A signal has disappeared in all the ICP0-expressing cells. Bars, 10 µm.

**Figure 5:**
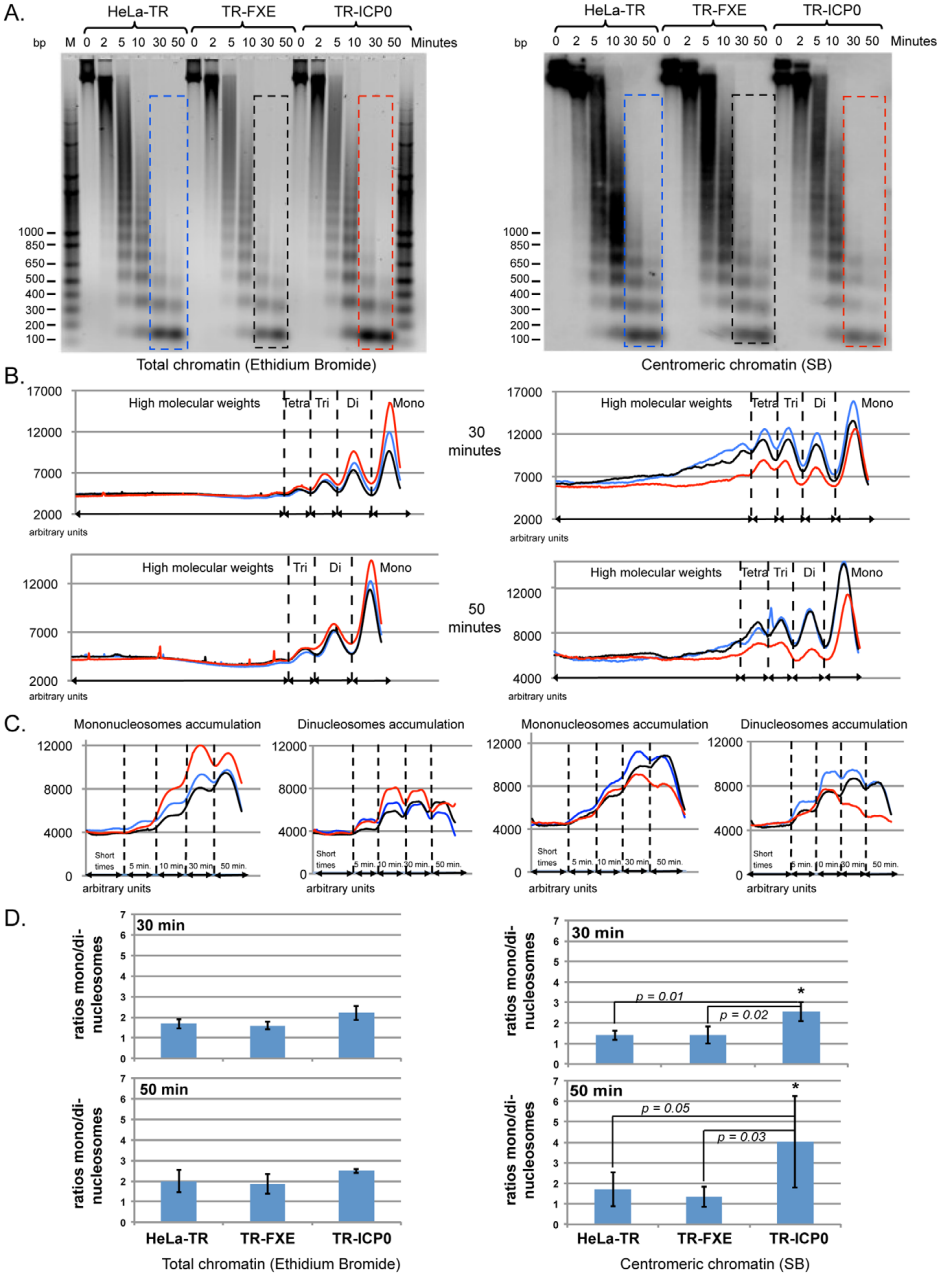
ICP0 alone destabilizes the centromere nucleosome structure. (A) Micrococcal nuclease (MNase) digestion patterns of the chromatin of three inducible cell lines: Control (no protein expressed) (HeLa-TR); expressing the ICP0 RING finger mutant FXE (TR-FXE); and expressing ICP0 (TR-ICP0). All cells were treated with tetracycline (1 µg/ml) for 19 h before the analysis. Left panel: MNase digestion patterns of total DNA. Right panel: Southern blot hybridization of the gel shown on the left using an alphoid DNA probe. M = 1 Kb Plus DNA ladder (in base pair, Invitrogen). (B) Plot of the digestion profiles after 30 min and 50 min of MNase digestion (curves corresponding to the colored frames in A). Red curve: HSV-1 wt infection; black curve, HSV-1 dl1403 infected cells; green curve: non-infected cells. (C) Quantification of mono- and di-nucleosomes during MNase digestion. Red curve: HSV-1 wt infection; black curve, HSV-1 dl1403 infected cells; green curve: non-infected cells. (D) Rate of chromatin digestion. The ratios of LMW-normalized mono- over di-nucleosomes (see Figure S4) give the rate of digestion of total chromatin (left panels) or centromeric chromatin (right panels). The graphs represent the mean values of the ratios of mono- over di-nucleosomes ± SD for at least three independent experiments. Results were considered as significant for a p value≤0.05 (Student's *t* test).

To confirm that ICP0 is directly involved in the destabilization of the centromeric chromatin structure, we performed MNase digestions of the chromatin from ICP0-expressing cells. We constructed HeLa cell lines that stably express ICP0 (TR-ICP0) or FXE (TR-FXE) following induction with tetracycline. Western blot analyzes showed that ICP0 or FXE is only expressed after induction (Figure 4A). We verified that ICP0 was functional in these cells, in that it efficiently induced the disappearance of CENP-A from interphase centromeres (Figure 4B, i to ix). No ICP0 was detected in the TR-ICP0 or TR-FXE cells before tetracycline induction (Figure 4B, ii and iii). After induction, >99.5% of the cells were positive for either ICP0 or FXE (Figure 4B, vi to ix). Only those cells that expressed ICP0 showed definite disappearance of the CENP-A signal (see left ICP0-expressing cell in Figure 4B, vi). These data confirm that the cell lines express ICP0 or FXE only when tetracycline is added to the medium, and that ICP0 induces the loss of CENP-A (and by extension, the other CENPs). MNase digestion of chromatin from non-induced HeLa-TR, TR-ICP0, and TR-FXE cells showed that there were no major differences in the LMW nucleosomal forms accumulation between the cell lines (data not shown). These cells were subsequently induced with tetracycline for 19 h, prior to MNase digestion. The results shown are representative of multiple experiments, and are presented in the same format as in Figure 3 with the blue, black, and red curves corresponding to HeLa-TR, TR-FXE, and TR-ICP0 cells respectively. Digestion of total cell chromatin showed that the accumulation of mononucleosomes was higher in the ICP0-expressing cells than in the HeLa-TR or TR-FXE cells (for 30 min and 50 min digestions; Figure 5A, 5B, and 5C left panels). Analysis of the appearance of HMW nucleosomal forms (octo- to penta-nucleosomes) did not show any obvious difference among HeLa-TR, TR-FXE and TR-ICP0 cells, which suggested that expression of FXE or ICP0 alone does not markedly modify the higher-order structure of the cellular chromatin (Figure S3). SB analysis of centromeric chromatin showed a deficit of LMW nucleosomal forms in the chromatin from the TR-ICP0 cells (for the 30 min and 50 min time points; Figure 5A and 5B, right panels), suggesting a faster digestion. This was confirmed by the analysis of the mono- and di-nucleosome accumulations over time. In TR-ICP0 cells unlike HeLa-TR and TR-FXE cells, signals of mono- and di-nucleosomes start decreasing at the end of experiment (from 50 min and 30 min, respectively) (Figure 5C, right panels). The rate of MNase digestion was evaluated as for the infected cells, using the LMW-normalized mono- and di-nucleosomes (Figure S4). The calculated ratios of mono- over di-nucleosomes in the ICP0-expressing cells at 30 min (2.5±0.5) and 50 min (4.0±2.2), were significantly higher compared to HeLa-TR cells (1.4±0.2 and 1.7±0.8 for 30 min and 50 min of digestion, respectively) and TR-FXE cells (1.4±0.4 and 1.4±0.5 for 30 min and 50 min of digestion, respectively) (Figure 5D). These data demonstrate that the expression of ICP0 alone is sufficient to destabilize centromeric chromatin.

### ICP0 induces iCDR on artificial centromeres

**Figure 6:**
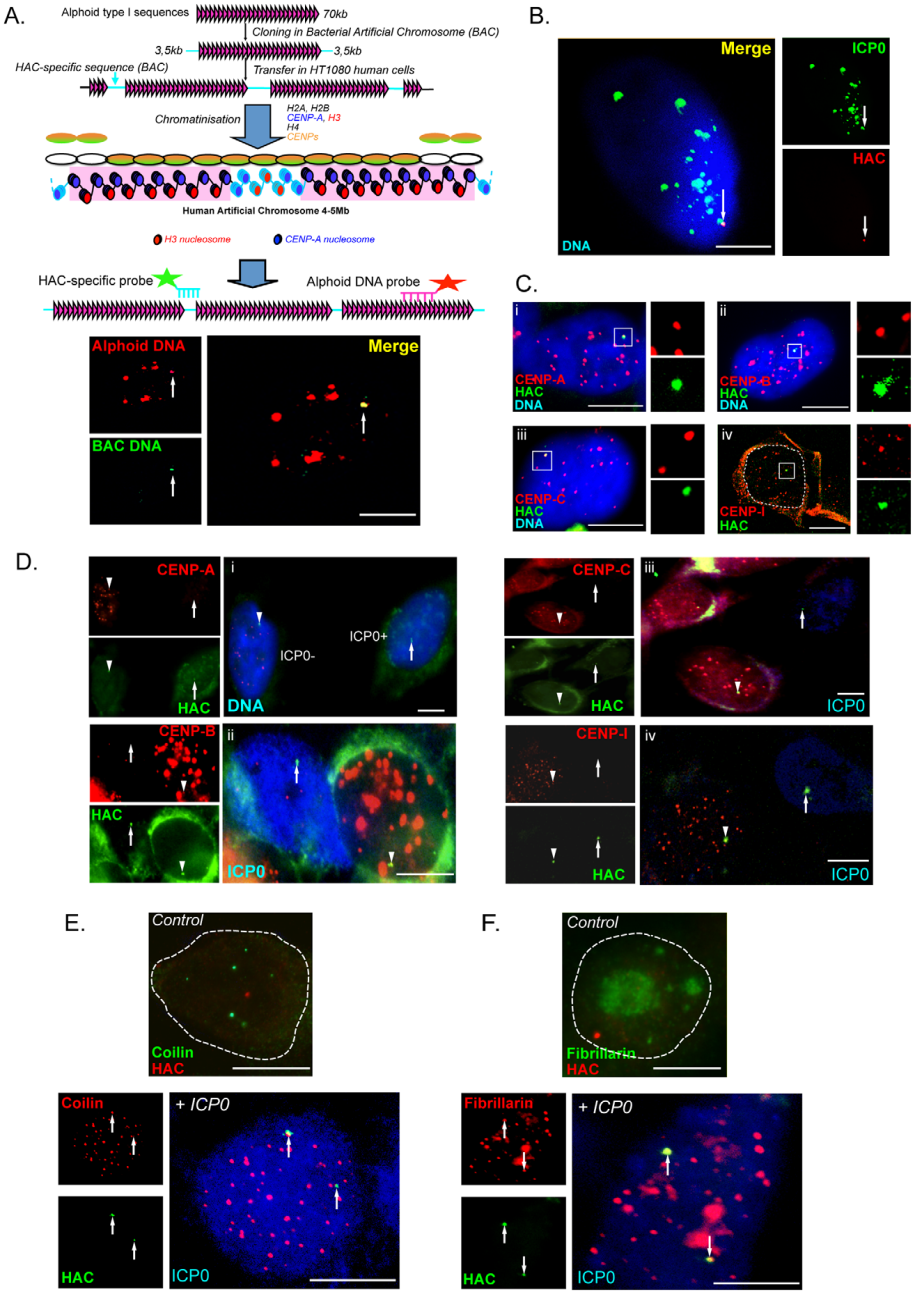
ICP0 triggers iCDR on human artificial chromosomes. (A) Upper panel, Formation and schematic structure of a human artificial chromosome (HAC). Lower panel, DNA-FISH showing the co-detection of HAC and alphoid DNA; the position of the probes for detection of HAC and alphoid DNA sequences are indicated above the images. Bar, 10 µm. (B) Immuno-DNA FISH showing the co-localization of ICP0 and endogenous centromeres in the interphase, different stages of mitosis, and on a mitotic chromosome (i to v). HT1080 W0210-R8 cells that contain a HAC were infected for 3 h with HSV-1 wt, and ICP0 and the HAC were detected (vi). Bars, 10 µm. (C) Immuno-DNA FISH showing the presence of CENP-A, -B, -C, and -I on the HAC in interphase cells. Images i, ii, and iii were obtained using a wide-field microscope, while image iv was acquired using a confocal microscope. The nucleus is outlined by a dashed line in iv. Bars, 10 µm. (D) Immuno-DNA FISH showing the disappearance of CENPs proteins from the HAC in HT1080 W0210-R8 cells infected for 5 h with the HSV-1 wt virus. DNA (i) or ICP0 (ii to iv) is visualized in blue. Arrowheads indicate HACs with CENPs co-localized (in ICP0- cells), while the arrows indicate HACs with no CENP signals (in ICP0+ cells). Bars, 10 µm. (E and F) Immuno-DNA FISH detection of coilin (E), fibrillarin (F), and HAC in HT1080 W0210-R8 cells infected with HSV-1 wt for 4 h. Coilin and fibrillarin are shown in control (not expressing ICP0) cells together with the HAC. Multiple spots of coilin and fibrillarin in ICP0-expressing cells are representative of the iCDR on endogenous centromeres in the ICP0-expressing cells (see [47]). Arrows indicate coilin or fibrillarin colocalizing with the HAC. Dotted lines in control images delimit the nucleus edge. Bars, 10 µm.

In human cells, in contrast to murine cells [51], the differentiation of centromeres from pericentromeres by immunocytochemical labeling is hindered by the spatial resolution of the signals that correspond to the two structures. As an example, simultaneous detection by classical confocal microscopy of heterochromatin protein 1 (HP1), which is a pericentromeric-associated protein, and centromeres gives co-localizing signals [52]. To confirm that the iCDR specifically targets destabilized centromeres, we analyzed the effect of ICP0 on HACs. HACs are independent, fully functional centromeres that exhibit mitotic stability comparable to endogenous chromosomes (for review [53]). HACs are made of alphoid sequences interspersed with BAC vector sequences, and thus are lacking canonical pericentromeric satellite repeats (Figure 6A). HACs are commonly used to study the structural and functional features of centromeres [54]–[57]. HT1080 cells that contained a HAC were initially assessed for the *in situ* detection of the HAC during interphase using both, a probe that detects the alphoid DNA, and a probe that is specific for the BAC sequences used to construct the HAC (see Materials and Methods; Figure 6A). To confirm that targeting of ICP0 to the HAC is similar to that observed for endogenous centromeres [30], cells were infected for 3 h with HSV-1 wt, and ICP0 and the HAC were detected by immuno-FISH. There was clear co-localization of the ICP0 and HAC signals (Figure 6B). The CENP-A, -B, -C and -I proteins were detected in non-infected cells, confirming the presence of proteins from the inner centromere, the NAC and CAD complexes, on the HAC (Figure 6C). We then analyzed the disappearance of these proteins from the HAC in infected (Figure 6D) and transfected (data not shown) cells. We found that all these CENPs disappeared from the HAC in the ICP0-expressing cells (Figure 6D). Analysis of the triggering of iCDR on the HAC revealed that coilin and fibrillarin, which in control (not expressing ICP0) cells do not colocalize with the HAC, were redistributed on the destabilized HAC (Figure 6E and F). These data confirmed that the iCDR is triggered in response to the destabilization of the centromere structure.

## Discussion

The present study demonstrates that the ICP0 protein of HSV-1 acts as a powerful modifier of centromeres. HSV-1, through the sole activity of ICP0, is able to induce the disappearance of several CENPs, which are components of the proteinaceous layers of interphase centromeres. ICP0 induces the proteasomal degradation of several CENPs, irrespective of whether they belong to the CAD or NAC complex. In addition, we show that the centromeric chromatin structure is greatly affected by ICP0 activity.

Proteins of the NAC and CAD complexes, also referred to as constitutive centromere associated network (CCAN), play essential roles in providing a suitable platform for kinetochore assembly. Recently, it has been demonstrated that CENP-C and CENP-T, which were described as components of the NAC, are crucial for the formation of the kinetochore in both human and *Drosophila* cells [58]–[61]. Moreover, CENP-C and CENP-N were shown to bind directly to CENP-A, but not to H3, -containing nucleosomes [62], [63]. CENP-N is a key subunit of the CCAN that is required for the loading of all other CCAN subunits onto kinetochores [15], [17], [62]. This study and previous ones show that ICP0 targets for proteasomal degradation several major components of the CCAN including CENP-H, CENP-I, CENP-N, and CENP-C [30], but also of the chromatin (CENP-A, [41]), and of the inner centromere (CENP-B, [42]). Therefore, the ICP0-induced loss of CAD-associated CENP-I, -O, -P, and -Q and NAC-associated CENP-C, -H, -N, and -M observed during infection or transfection of GFP-CENP-expressing cells is most likely reflecting the complete breakdown of the whole proteinaceous structure. Of the CENPs targeted by ICP0 for degradation, CENP-B and -C have been shown to bind directly alphoid DNA [64]–[68], and CENP-A is present in the heart of the nucleosome, replacing histone H3 [8], [69], [70]. This suggested that ICP0 anti-centromere activity could also destabilize the centromeric chromatin structure. MNase digestions of HSV-1-infected and ICP0-expressing cells confirmed that ICP0 induces significant modifications of the nucleosomal organization of centromeric chromatin. Previous studies showed that infection could affect cellular chromatin in an ICP0-independent manner [48]–[50]. Our MNase digestion data also show that infection alters cellular chromatin but the effect was restricted to the HMW nucleosomal forms. These observations indicate that HSV-1 infection modifies the higher-order chromatin fiber folding independently of ICP0, and does not significantly affect the structure of the nucleosome. Thus the effect we observed on LMW centromeric chromatin is not the consequence of the infection itself. Taken together, our data highlight the potent effects of ICP0 on centromere structure, and raise the possibility that HSV-1 has found a selective advantage in targeting centromeres.

Recently, we described a novel cellular response, named iCDR (for interphase Centromere Damage Response), which is triggered following ICP0-induced centromere destabilization [47]. The iCDR is conserved, at least in humans and mice, and involves the accumulation at damaged centromeres of coilin, fibrillarin, and survival motor neuron proteins, three major components of the nuclear domains Cajal bodies/Gemini bodies. The iCDR is not a cellular response to DNA damage, but is, rather, a consequence of centromeric chromatin modifications. Indeed, depletion of CENP-B, which is implicated in the positioning of nucleosomes, chromatin epigenetic modifications, and DNA methylation [56], [71], [72], also triggers coilin relocation at centromeres [47]. Therefore, ICP0-induced centromeric chromatin destabilization could induce epigenetic changes that stimulate the relocation of specific cellular proteins to the damaged centromere. Moreover, we have found that ICP0 is able to localize to artificial centromeres made from HACs and to induce the disappearance of HAC-associated CENPs. These observations support the notion that the targeting of ICP0 to centromeres is not a random process, and that components of centromere identity are sufficient. The fact that iCDR is triggered on HACs suggests that iCDR is a response involving destabilized centromeres rather than pericentromeres at least based on DNA sequences. In addition, we have evidence that the destabilization of HP1 proteins by siRNA does not trigger iCDR on pericentromeres (data not shown). Studies of endogenous centromeres are complicated due to the fact that they share DNA sequence similarities but are not identical. HACs are outstanding tools for studies on the structural and functional characteristics of centromeres, as they allow sequence-specific discrimination between HAC-associated centromeres and endogenous centromeres. Recent studies have established that HACs harbor epigenetic modifications found within endogenous centromeres [54]–[57]. If iCDR is somehow linked to epigenetic changes that result from centromeric chromatin destabilization, then HACs may prove to be valuable tools for analyzing the mechanism of iCDR.

To date, HSV-1 remains the only virus known to destabilize centromeres. The challenge is to understand the reasons for this particular activity. A simplistic explanation would be to evoke a side effect of the multiple ICP0 activities. This is highly unlikely, since several ICP0 homologs of other alphaherpesviruses have been shown to provoke CENP-C disappearance from centromeres, and the equine herpesvirus type 1 ICP0 homolog (Eg63) induces CENP-C proteasomal degradation when expressed from a recombinant HSV-1 virus [73]. If evolution has retained such an activity associated with proteins from different viruses, it is reasonable to propose that it plays a major role in the biology of these viruses. Another possibility is that HSV-1 targets a centromere-associated signaling pathway, indicating that the cell undergoes an undesirable event that, if not brought under control, could lead to cell death. The interaction of CENP-C with hDaxx, a protein involved in signaling apoptosis, lends some credibility to this hypothesis [74]. However, infection of cultured cells at high multiplicity of infection (m.o.i.) does not require ICP0 and subsequently centromere destabilization; and infection at low m.o.i. with an ICP0-deleted HSV-1 does not induce substantial apoptosis of infected cells. These two findings do not support the idea of a centromere-controlled cell fate, at least for cultured cells. ICP0 could induce centromere destabilization to provoke the mitotic arrest of infected cells [46]. However, this appears to contradict the known biology of the virus, as HSV-1 requires the cell nucleus for its replication. Moreover, HSV-1 replicates independently of the cell cycle, and the lytic cycle does not depend on cell arrest at the mitotic phase [75].

Therefore, ICP0-induced centromere inactivation needs to be analyzed in light of an activity of ICP0 not related to lytic infection. After an initial lytic infection in epithelial cells of the buccal mucosa or orofacial skin, HSV-1 establishes life-long latency in sensory neuron nuclei of the infected host. From the molecular point of view, latent HSV-1 DNA exhibits transcriptional shut down, with only a family of non-coding RNAs, called LATs (latency-associated transcripts) being generated [25], [76], [77]. Reactivation of the virus occurs following different stresses, and ICP0 has been shown to be essential for full reactivation in a mouse model [26], [27]. Using a fluorescence *in situ* hybridization (FISH) approach specifically developed to detect latent HSV-1 genomes within neuronal tissues, we have shown that HSV-1 genomes are not randomly distributed in the nuclei of infected neurons [78]. Interestingly, a significant proportion of the infected neurons displayed HSV-1 genomes that co-localized with centromeres [78]. These centromere-associated viral genomes were shown to be constantly negative for the expression of LATs. This means that during latency there is a close interplay between transcriptionally inactive HSV-1 genomes and centromeres. It is known that centromeres are preferential sites of inactive cellular gene deposition in differentiated cells, which could act to maintain heritable silencing of genomic loci [79]–[89]. Therefore, it is intriguing that repressed latent HSV-1 genomes are also found at centromeres, and that ICP0, which is essential for reactivation, destabilizes these domains. A reasonable hypothesis is that the ICP0 anti-centromere activity is required to induce optimal reactivation of HSV-1 from latency. We are currently testing this hypothesis.

## Materials and Methods

### Cells, viruses and plasmids

HeLa cells were grown at 37°C in Glasgow modified Eagle's medium (GMEM). HeLa cells that constitutively express GFP-CENP-H, -M, -N, -O, -P, and -Q (generous gifts from D. Foltz and I. Cheesman, Ludwig Institute for Cancer Research, San Diego; CA, USA), HeLa TREX (HeLa-TR; Invitrogen), TR-ICP0 (TR-ICP0.31.5, derived from TR-ICP0.39 [42], and TR-FXE (TR-FXE.7) cells were used (Commission de génie génétique : approval procedure n°4937). HT1080 cells [90] were grown in DMEM. All media were supplemented with 10% fetal bovine serum, L-glutamine (1% v/v), 10 U/ml penicillin, and 100 µg/ml streptomycin. For the HeLa-TR cells, blasticidin (5 µg/ml) was added to the medium, and for TR-ICP0 and TR-FXE cells, blasticidin and zeocin (100 µg/ml) were added. The TR-ICP0 and TR-FXE are tetracycline inducible cell lines, which express ICP0 and FXE (the non-functional ICP0 RING finger mutant, [91]), respectively. They were constructed by transfecting HeLa-TR cells with a pcDNA 4/TO plasmid (Invitrogen) that was designed to express ICP0 or FXE. Individual cell clones were isolated under zeocin selection. Expression of ICP0 or FXE was induced by the addition of tetracycline (1 µg/ml) to the medium, and checked by Western blotting and immunocytochemistry. Construction and validation of the GFP-CENP-expressing HeLa cell lines has been described previously [14]. HT1080 W0210-R8 cells, which contain a human artificial chromosome (HAC), have been described previously [90]. The wild type strain HSV-1 17syn+ (HSV-1 wt) was the parental strain used in the present study. The viruses dl1403, which is deleted for ICP0 [92], and vFXE, which expresses a non-functional ICP0 isoform mutated in the RING finger domain [91], were also used. The pci110 and pciFXE plasmids that express ICP0 and FXE were described previously [91].

### Micrococcal nuclease (MNase) digestion

MNase digestions were performed either on infected cells or HeLa-TR cells induced or not induced for the expression of ICP0 or FXE. Cells were seeded at 3×10^6^ cells per 10 cm Petri dish 24 h before the experiment. HeLa cells were infected with the appropriate virus at a multiplicity of infection (m.o.i.) of 10 (all cells infected) for 3 h. HeLa-TR, TR-ICP0 and TR-FXE were induced with tetracycline (1 µg/ml) or left untreated for 24 h. The cells were trypsinized, washed with PBS, centrifuged, and re-suspended in 1 ml of Buffer 1 (15 mM Tris-HCl [pH 7.5], 0.3 M sucrose, 60 mM KCl, 15 mM NaCl, 5 mM MgCl_2_, 0.1 mM EGTA, 0.5 mM DTT, and protease inhibitors). Then, 1 ml of Buffer 2 (15 mM Tris-HCl [pH 7.5], 0.3 M sucrose, 60 mM KCl, 15 mM NaCl, 5 mM MgCl_2_, 0.1 mM EGTA, 0.5 mM DTT, 0.4% NP40, and protease inhibitors) was added gently and the cells were incubated at 4°C for 10 min. Thereafter, 8 ml of Buffer 3 (15 mM Tris-HCl [pH 7.5], 1 M sucrose, 60 mM KCl, 15 mM NaCl, 5 mM MgCl_2_, 0.1 mM EGTA, 0.5 mM DTT, and protease inhibitors) were then added at the bottom of the tube, and the tube was centrifuged for 30 min at 6000× *g* at 4°C. The supernatant was removed gently, and the cell nuclei were resuspended in MNase digestion buffer (15 mM Tris-HCl [pH 7.5], 5 mM MgCl_2_, 1 mM CaCl_2_, and protease inhibitors) at an estimated concentration of 1000 nuclei/µl in a volume of 3 ml. The nuclei were pre-incubated for 2 min at 37°C, and 5 U of MNase per 3×10^6^ nuclei were added. At fixed time points, 3.75×10^5^ nuclei were harvested, EDTA was added to a final concentration of 0.8 mM to stop the reaction, and the cells were kept on ice. Subsequently, protein digestion buffer (10% SDS, 3.75 µg/µl proteinase K) was added at 1∶20 to the samples, which were incubated for 15–30 minutes at 37°C. DNA was recovered by phenol/chloroform extraction followed by precipitation with ethanol. The DNA was re-suspended in water containing RNase A (1 µg/ml) and incubated for 1 h at 37°C.

### Southern blotting and probe labeling

DNA (1 µg/lane) was electrophoresed for 17 h at 20 V in a 1.2% agarose gel in 0.5× TAE with 1 mg/ml ethidium bromide, transferred onto a nylon membrane (Hybond N+; GE Healthcare) using a vacuum apparatus, and UV-crosslinked. The 1.9-kb probe used to detect the centromeric repetitive DNA (alphoid sequences) was amplified by PCR (with primers M13U: 5′-gttgtaaaacgacggcc-3′; and M13R: 5′-caggaaacagctatgac-3′) from the plasmid p11-4 containing the alphoid centromeric sequence of human chromosome 21 [93]. The amplified alphoid DNA (50 ng) was labeled by random-priming (Ready-To-Go DNA labeling beads; GE Healthcare), together with 50 µCi of [^32^P] dCTP (Hartman Analytic) and purified on a G50 column (GE Healthcare). The membrane was pre-hybridized in the hybridization solution (Ambion) for 1 h at 44°C. The probe was denatured with 1 mg of salmon sperm DNA. Hybridization was performed for 16 h at 44°C. Washing was performed in SSC-0.5% SDS at 65°C. Hybridization was detected using a Typhoon 9400 system (GE Healthcare), and the signals were quantified, ImageJ software (http://imageJ.nih.gov/ij/).

### Fluorescent in situ hybridization (FISH)

FISH was adapted from [94]. Cells were grown on coverslips. The cells were fixed with 2% PFA and permeabilized with 0.5% Triton X-100 in PBS. Probe accessibility to DNA was obtained by heat-based unmasking in 100 mM citrate buffer, and DNA deproteination in 0.1 M HCl for 5 min. Hybridization mix contained 40 to 100 ng of probe in 10% dextran, 1× Denhardt's, 2× SSC, 50% formamide. DNA denaturation was performed for 5 min at 80°C, and hybridization was carried out overnight at 37°C. The cells were washed by incubation in 2× SSC and 0.2× SSC at 37°C. Detection of biotin-labeled probes was performed with AlexaFluor 488-conjugated streptavidin diluted in 4× SSC/5% fat-free milk. Nuclei were stained with Hoechst 33342 (Invitrogen). The coverslips were mounted using Vectashield mounting medium (Vector Laboratories) and stored at 4°C until observation.

### Probe labeling

The alphoid sequence was detected with the same probe used for southern blot. The HAC was detected with a probe corresponding to the BAC sequence, derived from a 4.66-kb fragment amplified from the pWTR11.32 plasmid [90]. The following primers were used: BACS, 5′-gctcgtcgacagcgacacacttgcatcgg-3′; and BACX, 5′-ccctcgagtgagcgaggaagcaccaggg-3′. The probes were labeled with dCTP-cy3, dCTP-cy5 or dUTP-biotin by nick-translation (Roche). The labeled DNA was purified on G50 columns (GE Healthcare), and the DNA was precipitated with ethanol and resuspended in formamide.

### Immunofluorescence and immuno-DNA FISH

For immunofluorescence (IF), cells were grown on a round coverslip and treated as described previously [47]. For immuno-DNA FISH, the cells were treated as described for the FISH protocol up to the unmasking step, and then immunostained. Following immunostaining, the cells were post-fixed in 1% PFA, and DNA-FISH was carried out from the HCl step. Imaging was performed using an inverted CellObserver (Zeiss) and a CoolSnap HQ2 camera from Molecular Dynamics (Ropper Scientific) and a Zeiss LSM 510 confocal microscope (Zeiss) using a PlanApochromat ×100, N.A. 1.3 objective. Datasets were processed using the LSM 510 software and ImageJ.

### Western blotting (WB)

HeLa cells (5×10^5^) grown in 35-mm Petri dishes were infected for 6 h at an m.o.i. of 10 (i.e., all cells infected) with HSV-1 wt, vFXE or dl1403, in the presence or absence of the proteasome inhibitor MG132 (2.5 µM). For the HeLa-TR, TR-ICP0, and TR-FXE lines, the cells were seeded at 1×10^6^ cells per 60-mm Petri dish, and the following day tetracycline was added, or not, to the medium for 24 h. Aliquots (10 µg) of total protein were electrophoresed in an SDS-polyacrylamide gel, before transfer and detection.

### Antibodies

The following antibodies were used for IF and WB: monoclonal antibodies anti-ICP0 (clone [11060], 1/1000 for IF; 1/10000 for WB; a kind gift from R. D. Everett, CVR-Glasgow, UK), anti-CENP-A (clone [3]–[19] 1 µg/ml for IF and WB; Abcam), and anti-CENP-B (clone [5E6C1]; 1/2000); rabbit polyclonal antibodies (r554) against CENP-C (a kind gift from W. C. Earnshaw); rabbit polyclonal antibodies anti-CENP-I (1/1000, for IF; 1 µg/ml for WB; Abcam); anti-CENP-N (Chl4R) (1/200 for WB: a kind gift from P. Meraldi, ETH Zurich, Switzerland), anti-CENP-H (1/100; a kind gift from K. Todokoro, RIKEN, Japan); anti-actin antibody (1 µg/ml for WB; Sigma); and a human autoimmune serum huACA against centromeric proteins (1/3000 for IF; Antibodies Incorporated). For IF, the secondary antibodies used were: goat anti-rabbit, anti-mouse, and anti-human coupled to Alexa Fluor 488, Alexa Fluor 555, and Alexa Fluor 647, respectively (1/200; Molecular Probes).

### Statistical analysis

Student's *t*-tests were performed using Microsoft Excel version 14.2.3 for MAC OS X. The results were calculated with the paired test. Results were considered as significant for a p value≤0.05.

## Supporting Information

Figure S1 **Kinetic of HMW forms appearance in total chromatin from infected cells.** HMW forms (from octo- to penta-nucleosomes) were quantified at 2, 5, 10, 30 and 50 min of MNase digestion in mock (blue), dl1403 (black) and HSV-1 wt (red)-infected cells. Peak of accumulation of each form over time correlates with the accessibility of the total chromatin to the MNase. Chromatin digestion peak in dl1403 and HSV-1 wt-infected cells precedes that of mock-infected cells suggesting a better accessibility of the higher-order chromatin structure to produce the HMW in infected cells, and in an ICP0-independent manner.
(PDF)

Figure S2 **Ratios of mono- and di-nucleosomes normalized on LMW in infected cells.** Relative abundances of mono- (blue) and di-nucleosomes (red) among the four lowest molecular weight forms (mono-, di-, tri-, and tetra-nucleosomes) at 30 min and 50 min of MNase digestion in mock, dl1403 and HSV-1 wt infected cells were calculated from three independent experiments.
(PDF)

Figure S3 **Kinetic of HMW forms appearance in total chromatin from tetracyclin-induced cells.** HMW forms (from octo- to penta-nucleosomes) were quantified at 2, 5, 10, 30 and 50 min of MNase digestion in HeLa-TR, TR-FXE, and TR-ICP0 cells. Peak of accumulation of each form over time correlates with the accessibility of the total chromatin to the MNase.
(PDF)

Figure S4 **Ratios of mono- and di-nucleosomes over LMW in tetracyclin-induced cells.** Relative abundances of mono- (blue) and di-nucleosomes (red) among the four lowest molecular weight forms (mono-, di-, tri-, and tetra-nucleosomes) at 30 min and 50 min of MNase digestion in HeLa-TR, TR-FXE and TR-ICP0 tetracyclin-induced cells were calculated from three independent experiments.
(PDF)
