# Supplementary figures and images for: Centromere Architecture Breakdown Induced by the Viral E3 Ubiquitin Ligase ICP0 Protein of Herpes Simplex Virus Type 1
Source: PLoS One. 2012 Sep 20;7(9):e44227. doi: 10.1371/journal.pone.0044227 (PMC3447814; doi:10.1371/journal.pone.0044227)

**Octo-nucleosomes**

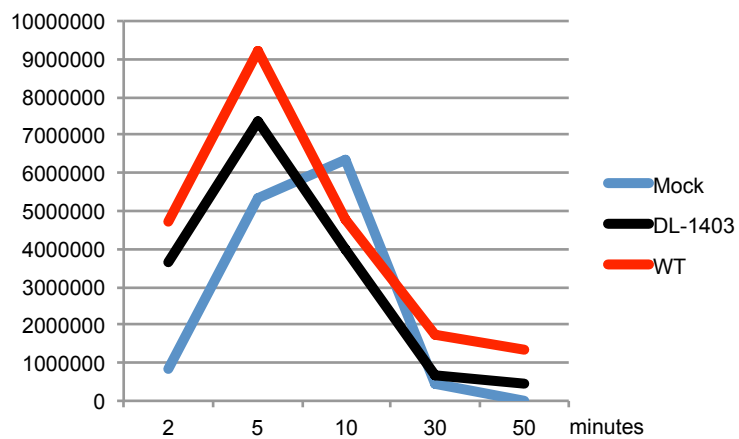

**Hepta-nucleosomes**

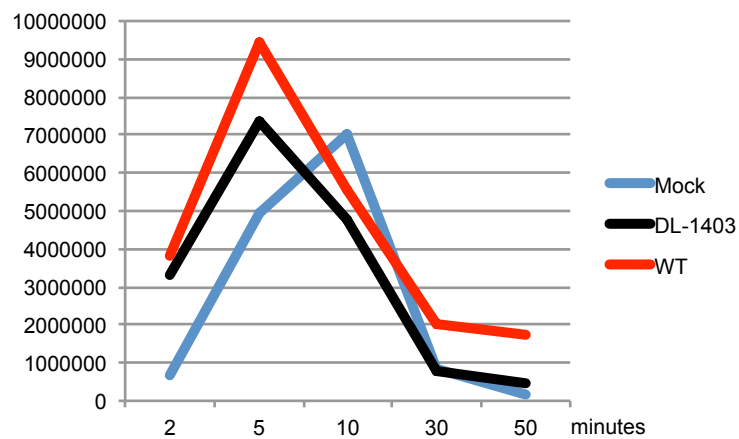

**Hexa-nucleosomes**

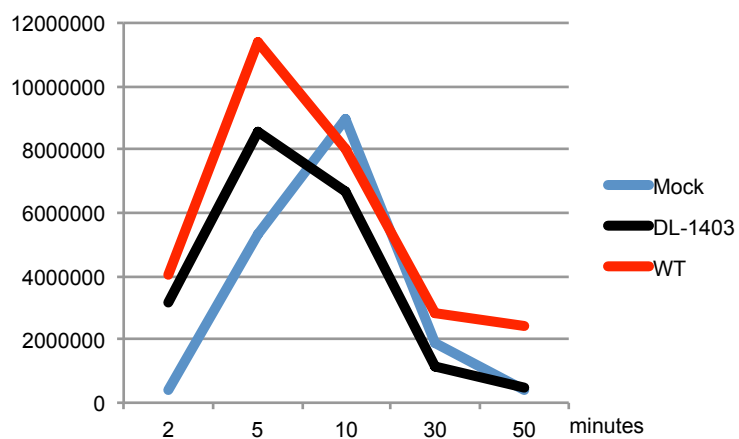

**Penta-nucleosomes**

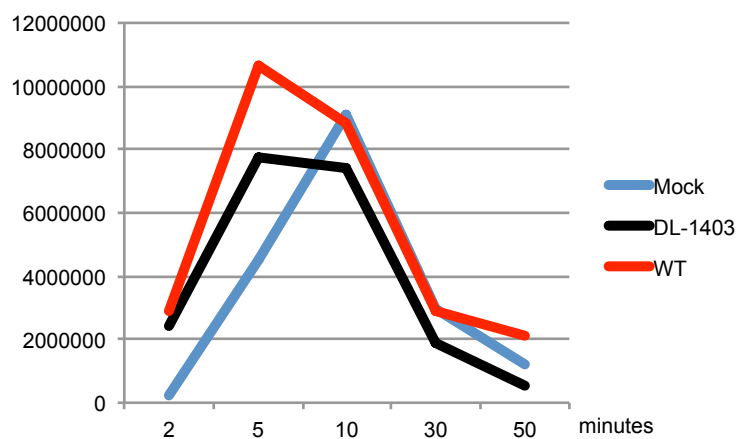

FIGURE S1- Gross *et al.*

Supplement: Figure S1 — Kinetic of HMW forms appearance in total chromatin from infected cells. HMW forms (from octo- to penta-nucleosomes) were quantified at 2, 5, 10, 30 and 50 min of MNase digestion in mock (blue), dl1403 (black) and HSV-1 wt (red)-infected cells. Peak of accumulation of each form over time correlates with the accessibility of the total chromatin to the MNase. Chromatin digestion peak in dl1403 and HSV-1 wt-infected cells precedes that of mock-infected cells suggesting a better accessibility of the higher-order chromatin structure to produce the HMW in infected cells, and in an ICP0-independent manner. (PDF) [file pone.0044227.s001.pdf]

**Octo-nucleosomes**

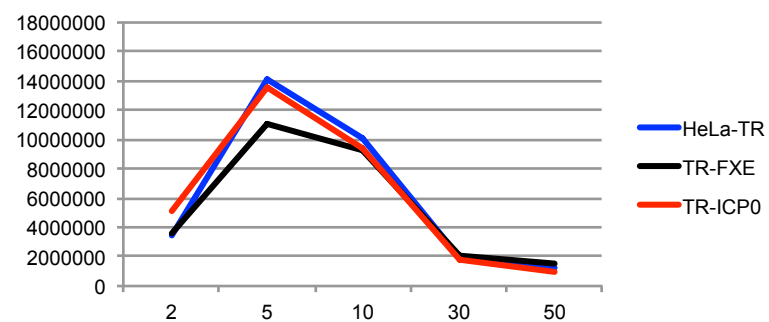

**Hepta-nucleosomes**

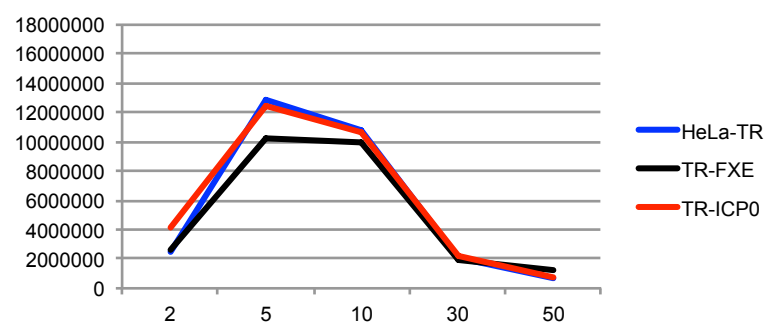

**Hexa-nucleosomes**

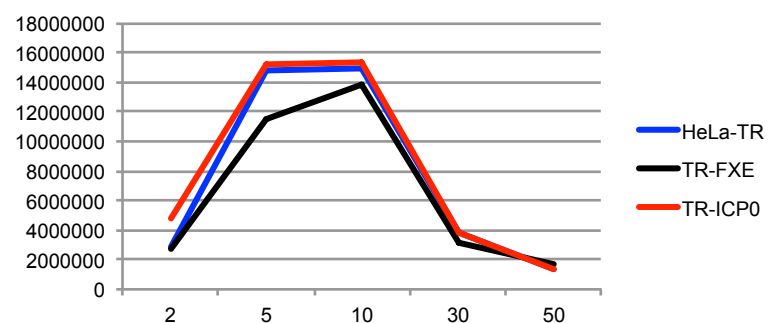

**Penta-nucleosomes**

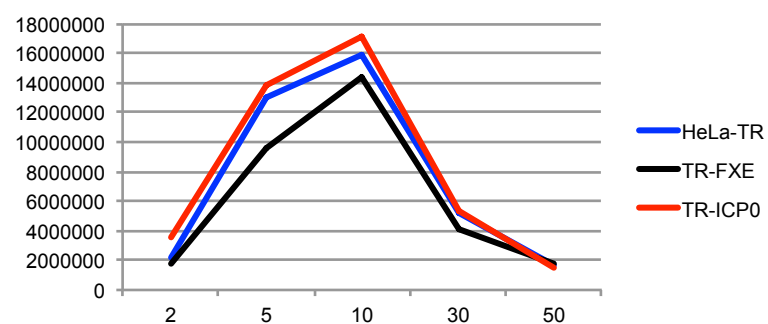

**FIGURE S3- Gross *et al.***

Supplement: Figure S3 — Kinetic of HMW forms appearance in total chromatin from tetracyclin-induced cells. HMW forms (from octo- to penta-nucleosomes) were quantified at 2, 5, 10, 30 and 50 min of MNase digestion in HeLa-TR, TR-FXE, and TR-ICP0 cells. Peak of accumulation of each form over time correlates with the accessibility of the total chromatin to the MNase. (PDF) [file pone.0044227.s003.pdf]

Total chromatin (Ethidium Bromide)

Centromeric chromatin (SB)

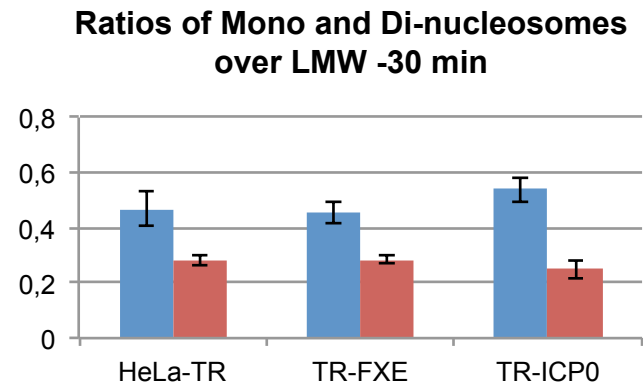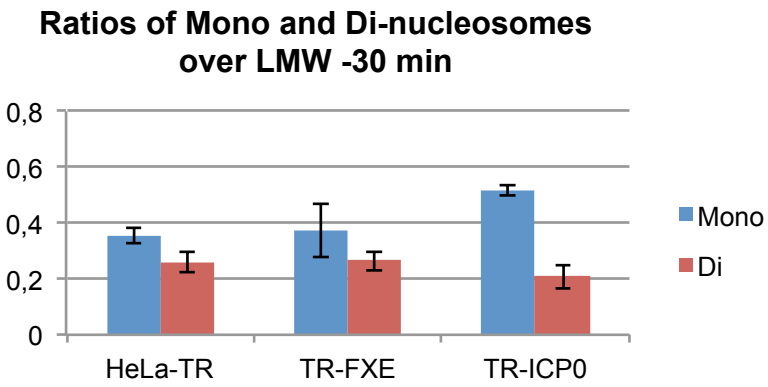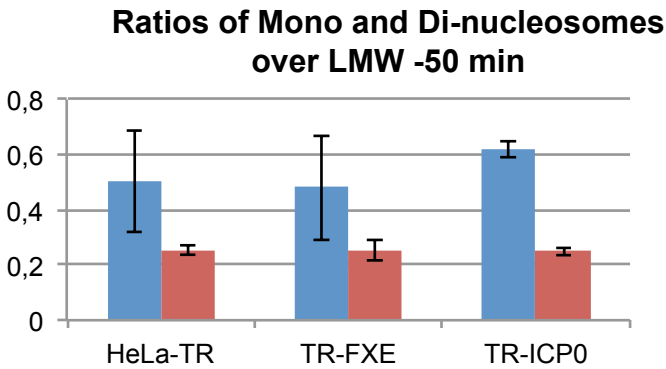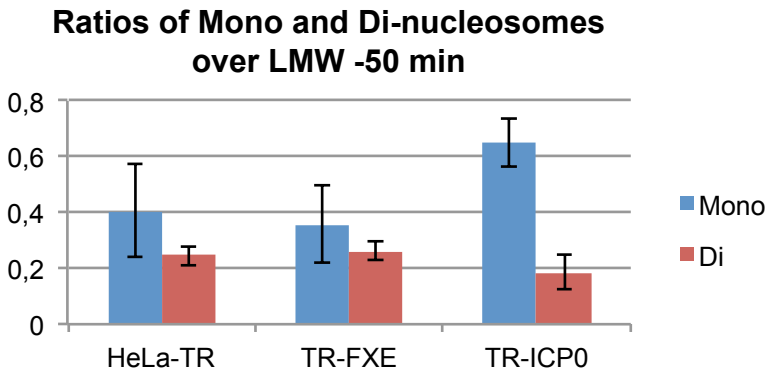

FIGURE S4- Gross *et al.*

Supplement: Figure S4 — Ratios of mono- and di-nucleosomes over LMW in tetracyclin-induced cells. Relative abundances of mono- (blue) and di-nucleosomes (red) among the four lowest molecular weight forms (mono-, di-, tri-, and tetra-nucleosomes) at 30 min and 50 min of MNase digestion in HeLa-TR, TR-FXE and TR-ICP0 tetracyclin-induced cells were calculated from three independent experiments. (PDF) [file pone.0044227.s004.pdf]
