# Supplementary material for: Centromere Architecture Breakdown Induced by the Viral E3 Ubiquitin Ligase ICP0 Protein of Herpes Simplex Virus Type 1
Source: PLoS One. 2012 Sep 20;7(9):e44227. doi: 10.1371/journal.pone.0044227 (PMC3447814; doi:10.1371/journal.pone.0044227)
Supplement: Figure S2 — Ratios of mono- and di-nucleosomes normalized on LMW in infected cells. Relative abundances of mono- (blue) and di-nucleosomes (red) among the four lowest molecular weight forms (mono-, di-, tri-, and tetra-nucleosomes) at 30 min and 50 min of MNase digestion in mock, dl1403 and HSV-1 wt infected cells were calculated from three independent experiments. (PDF) [file pone.0044227.s002.pdf]

Total chromatin (Ethidium Bromide)

Centromeric chromatin (SB)

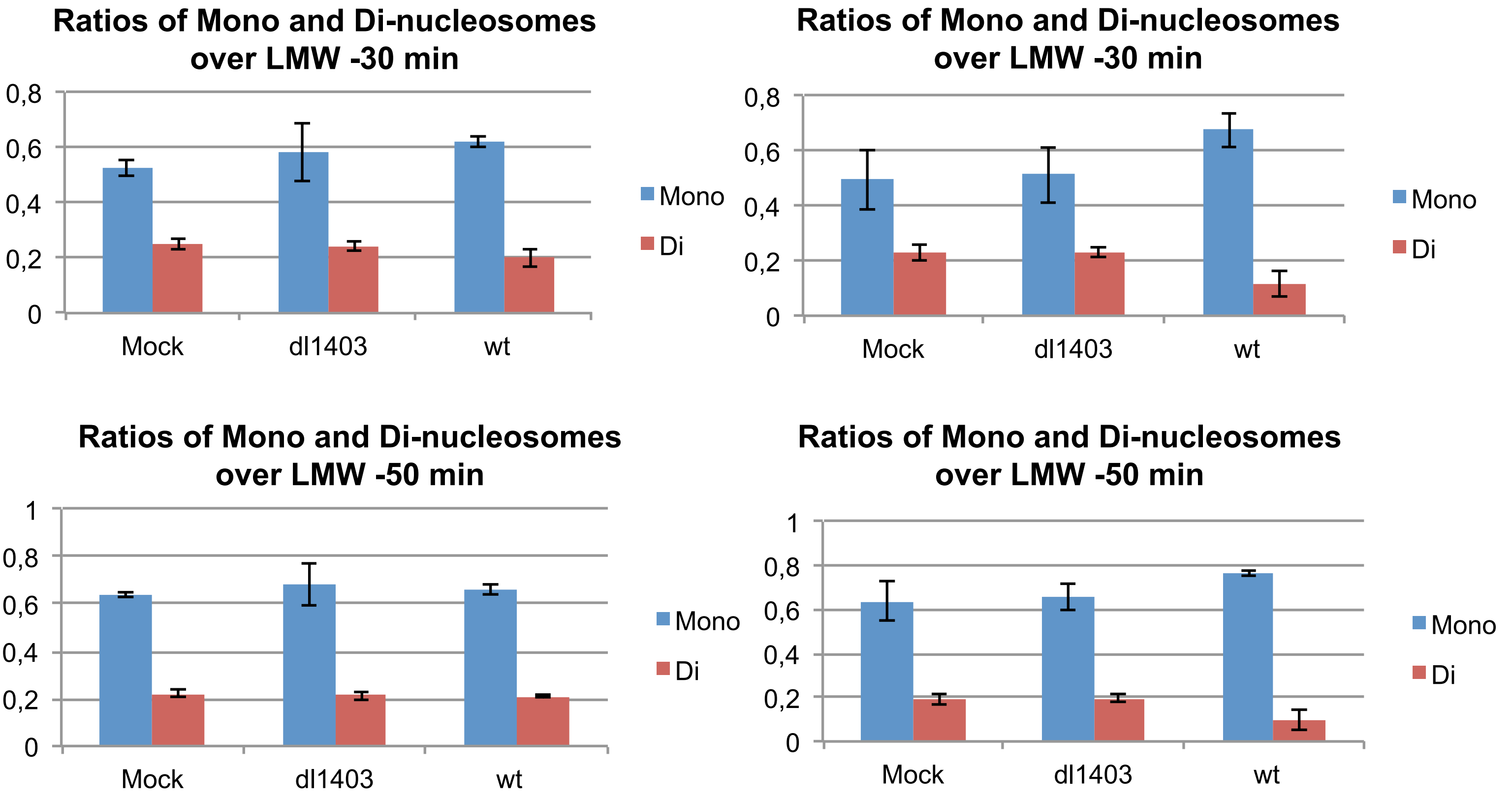

FIGURE S2- Gross *et al.*
